# Genome Sequences of Novel Azospirillum sp. Strains B21 and Sh1, Isolated from Raised Sphagnum Bogs, and Type Strains Azospirillum lipoferum 59b and Azospirillum oryzae COC8

**DOI:** 10.1128/MRA.01174-19

**Published:** 2019-10-24

**Authors:** Denis S. Grouzdev, Ekaterina N. Tikhonova, Irina K. Kravchenko

**Affiliations:** a Institute of Bioengineering, Research Center of Biotechnology of the Russian Academy of Sciences, Moscow, Russia; b Winogradsky Institute of Microbiology, Research Center of Biotechnology of the Russian Academy of Sciences, Moscow, Russia; University of Southern California

## Abstract

Here, we report the genomic sequences of the novel *Azospirillum* sp. strains B21 and Sh1, isolated from raised bogs, along with the genome sequences of Azospirillum lipoferum 59b^T^, the type species of the genus, and Azospirillum oryzae COC8^T^, which were analyzed to get more knowledge about the genus *Azospirillum*.

## ANNOUNCEMENT

Bacteria of the genus *Azospirillum*, belonging to the order *Rhodospirillales* and the family *Rhodospirillaceae*, are well-characterized plant growth-promoting rhizobacteria (PGPR) due to their capacity for fixing atmospheric nitrogen and their ability to colonize roots of cereals and other grasses and to produce phytohormones. Recently Azospirillum palustre, a novel methylotrophic bacterium isolated from raised bog, was described (1).

*Azospirillum* sp. strains B21 and Sh1 were isolated from acidic *Sphagnum*-dominated peat soil in the Russian Federation. Strain B21 was isolated from the Sosvyatskoye peatland in the Tver region in 2000 (2). Strain Sh1 was isolated in 2018 from the upper 0- to 5-cm layer of a typical raised bog in the Shatura region, Moscow Oblast, which was afforested, i.e., drained and planted with trees. Pure cultures were obtained by serial 10-fold dilutions on N-free NFb agar medium (1.5%) (3). The plates were incubated at 29°C for 72 h, and the isolates were kept as pure cultures. DNA was purified from the cell biomass using the DNeasy PowerSoil kit (Qiagen, Germany) following the manufacturer’s instructions. The 16S rRNA gene was amplified with the 27F and 1492R primers (4), and purified PCR products were sequenced with an ABI Prism 3730 DNA analyzer (Applied Biosystems, USA). The 16S rRNA sequence analysis conducted using the online tool EzBioCloud (5) revealed that B21 shares 99.2% similarity with Azospirillum oryzae COC8^T^ (6), and Sh1 shares 99.0% similarity with *Azospirillum palustre* B2^T^ (1, 7). Both bacteria were assigned to clade L (Azospirillum lipoferum) (8).

Despite the fact that Azospirillum lipoferum 59b^T^ (9) and Azospirillum oryzae COC8^T^ (6) have been described for a long time, genome-wide studies for these organisms were absent, which prevented a detailed comparison with newly isolated azospirilla. The strains Azospirillum lipoferum 59b^T^ (IBPPM 173 = VKM B-1519 = ATCC 29707) and Azospirillum oryzae COC8^T^ (IBPPM 548 = LMG 23844 = IAM 15130) were provided by the Collection of Rhizosphere Microorganisms (collection.ibppm.ru) in the framework of scientific cooperation.

To perform whole-genome sequencing, all strains were cultivated on the NFb agar medium for 72 h at 29°C. Colonies were washed from the plate with liquid NFb medium and centrifuged. The genomic DNA was extracted using the DNeasy PowerSoil kit (Qiagen, Germany), following the manufacturer’s instructions. The libraries were constructed with the NEBNext DNA library prep reagent set for Illumina according to the protocol for the kit. The genomes were sequenced using the HiSeq 2500 platform (Illumina, Inc., USA) with 150-bp (Azospirillum lipoferum 59b^T^, Azospirillum oryzae COC8^T^, and *Azospirillum* sp. strain Sh1) and 100-bp (*Azospirillum* sp. strain B21) paired-end reads. Low-quality reads were trimmed using Trimmomatic v. 0.36 (10) with the default settings for paired-end reads. Subsequently, the quality-filtered reads were *de novo* assembled with SPAdes v. 3.13.0 using the default settings (11). The resulting assemblies were quality assessed with QUAST v. 5.0 (12). Identification of protein-coding sequences and primary annotation were performed using the NCBI Prokaryotic Genome Annotation Pipeline (PGAP) (13). Summary statistics and characteristic features of the whole-genome sequencing, assembly, and annotation of the four strains are given in Table 1. These genome sequences provide valuable data to study the ecology, evolution, and physiology of *Azospirillum* species.

**TABLE 1:** Characteristics of draft genome sequences and accession numbers of Azospirillum oryzae COC8^T^, Azospirillum lipoferum 59b^T^, and *Azospirillum* sp. strains Sh1 and B21

	Data for strain:			
Characteristic	*A. oryzae* COC8^T^	*A. lipoferum* 59b^T^	*Azospirillum* sp. Sh1	*Azospirillum* sp. B21
BioProject no.	PRJNA563036	PRJNA563039	PRJNA562938	PRJNA563053
GenBank accession no.	VTTM00000000	VTTN00000000	VTTK00000000	VTTO00000000
BioSample no.	SAMN12661881	SAMN12661906	SAMN12660160	SAMN12662157
SRA no.	SRR10092265	SRR10092045	SRR10092256	SRR10103333
Genome size (bp)	6,755,201	7,987,183	7,274,603	7,463,459
G+C content (%)	67.36	67.27	67.71	67.33
No. of scaffolds	39	72	103	57
No. of pair-end reads	4,606,976	4,476,896	4,770,146	23,572,400
*L*_50_ value	5	7	14	6
*N*_50_ value (bp)	405,582	372,840	158,668	465,063
Coverage (×)	154	127	141	236
Total no. of genes	6,077	7,152	6,561	6,781
No. of protein-coding genes	5,859	6,903	6,319	6,534
No. of RNAs	74	76	74	76

### Data availability.

These whole-genome projects have been deposited at DDBJ/ENA/GenBank under the accession numbers listed in Table 1. The versions described in this paper are the first versions.
